# Neuroplasticity Improves Bipolar Disorder: A Review

**DOI:** 10.7759/cureus.11241

**Published:** 2020-10-29

**Authors:** Arohi B Gandhi, Ifrah Kaleem, Josh Alexander, Mohamed Hisbulla, Vishmita Kannichamy, Ishan Antony, Vinayak Mishra, Amit Banerjee, Safeera Khan

**Affiliations:** 1 Internal Medicine, California Institute of Behavioral Neurosciences & Psychology, Fairfield, USA

**Keywords:** brain and bipolar disorder, neuroplasticity, synaptic plasticity, neuroplasticity in bipolar, parietal lobe in bipolar, frontal lobe in bipolar, neuroimaging

## Abstract

Bipolar disorder (BD) is known for impairments in neurotrophic and neuroprotective processes, which translate into emotional and cognitive deficits affecting various brain regions. Using its neuroplastic properties, lithium, thus far, is the mood stabilizer used to amend the pathophysiological imbalance in BD. Neuroplasticity has gained massive popularity in the research department in the past decade, yet it lacks direct effort in changing the protocol through which physicians treat BD. Physical activity alongside cognitive therapy is theorized to produce long-term changes in the executive control network due to the assimilation of new neurons, amendment of emotional lability through hippocampal neurogenesis, and strengthening the stability of frontosubcortical and prefrontolimbic brain regions via neurogenesis. This review aims to provide an incentive for utilizing neuroplastic mechanisms concerning impairments dispensed by BD.

## Introduction and background

In the words of Max Cynader, "Neurons that wire together, fire together." Neuroplasticity is a process by which neurons undergo adaptive changes in response to challenges from the environment. The process by which this can be achieved is as follows: an environmental challenge will stimulate synaptic activity and membrane depolarization, which encourages receptor trafficking and neurotransmitters' release [1]. Increased synaptic activity between neurons creates new pathways and strengthens the existing, enhancing behavioral and cognitive performance. Neuroprogression in bipolar disorder (BD) is associated with damage in neuronal circuits leading to impaired neural plasticity, increased apoptosis along with discrepancies in synaptic transmission [2]. Neuroplasticity is associated with proliferating precursor cells, thus stimulating the survival of immature neurons [3]. Convergent evidence indicates neuroplasticity can be induced by various methods like physical activity [3], emotional and cognitive learning [1], lithium [4], natural-enriched environment [5], repetitive transcranial magnetic stimulation (rTMS) [6], magnetic seizure therapy (MST) [7], as well as enhancement of astrocytic plasticity [8], emotional resilience through effort-based reward (EBR) training [9], and brain-derived neurotrophic factor (BDNF) and peripheral vascular endothelial growth factor (VEGF) [10].

Bipolar disorder can be classified into two major categories: BD type I and BD type II. BD type I is associated with at least one manic episode, characterized by excessive activity, libido, and grandiose thinking followed by hypomanic or major depressive episodes [1]. In comparison, BD type II entails at least one depressive and one hypomanic episode, but no manic episodes. The parietal lobe (PL) is heavily involved in BD progression, as it supports the brain's cognitive functions, attention, and memory in particular [11]. Significantly altered functional connectivity in the executive control network (ECN) between the insula and the parietal lobule has been shown to differentiate between bipolar depression and unipolar depression [12]. Studies have demonstrated impaired attentional processing in patients with symptomatic bipolar disorder suggesting temporoparietal disturbances [13]. The frontal lobe is considered the control panel of an individual's personality due to its involvement in judgment, emotional expression, problem-solving, and memory. Studies have indicated the compromised integrity of frontosubcortical and prefrontolimbic brain regions with underlying dysregulation of glial-neuronal interactions in BD [4]. Hyper-perfusion of frontotemporal regions during emotional modulation suggests over-activation of the regions mentioned above has been seen in BD [14].

Despite extensive research, the etiology of bipolar disorder remains unresolved, but genetic and environmental factors have been successfully associated with BD development [2]. Neuroplasticity previously thought to remain dormant in the adult neocortex can be reactivated by sensory-motor interactions, in turn altering the pattern of neuronal connections [15]. Neuroplasticity is an expansive and hopeful concept which no one wants to stand against [13]. It has even been theorized to play a role in producing antidepressant-like effects via herbal medicine [16]. Neuroplasticity was first mentioned by Santiago Ramón y Cajal (1852-1934), known as the "father of neuroscience," and has only gained rapid momentum in the past decade [10]. The goal of this review is to investigate the beneficial effects of neuroplasticity in a BD brain.

## Review

Methods

An expansive search for published literature was performed using PubMed and Google Scholar to distinguish factors through which neuroplasticity can be enhanced in the brains of patients diagnosed with bipolar disorder. The search terms included were brain and bipolar disorder, neuroplasticity, synaptic plasticity, neuroplasticity in bipolar, parietal lobe in bipolar, frontal lobe in bipolar, and neuroimaging. Articles determined by the author, which focused on neuroplasticity's effect on the adult brain and specifically on a bipolar brain, were then examined in substantial depth and included in this review. Due to limited research on this subject, no exclusion was applied based on the study's age. Literature directly relevant to bipolar disorder and neuroplasticity was included. 

Results 

**Figure 1:**
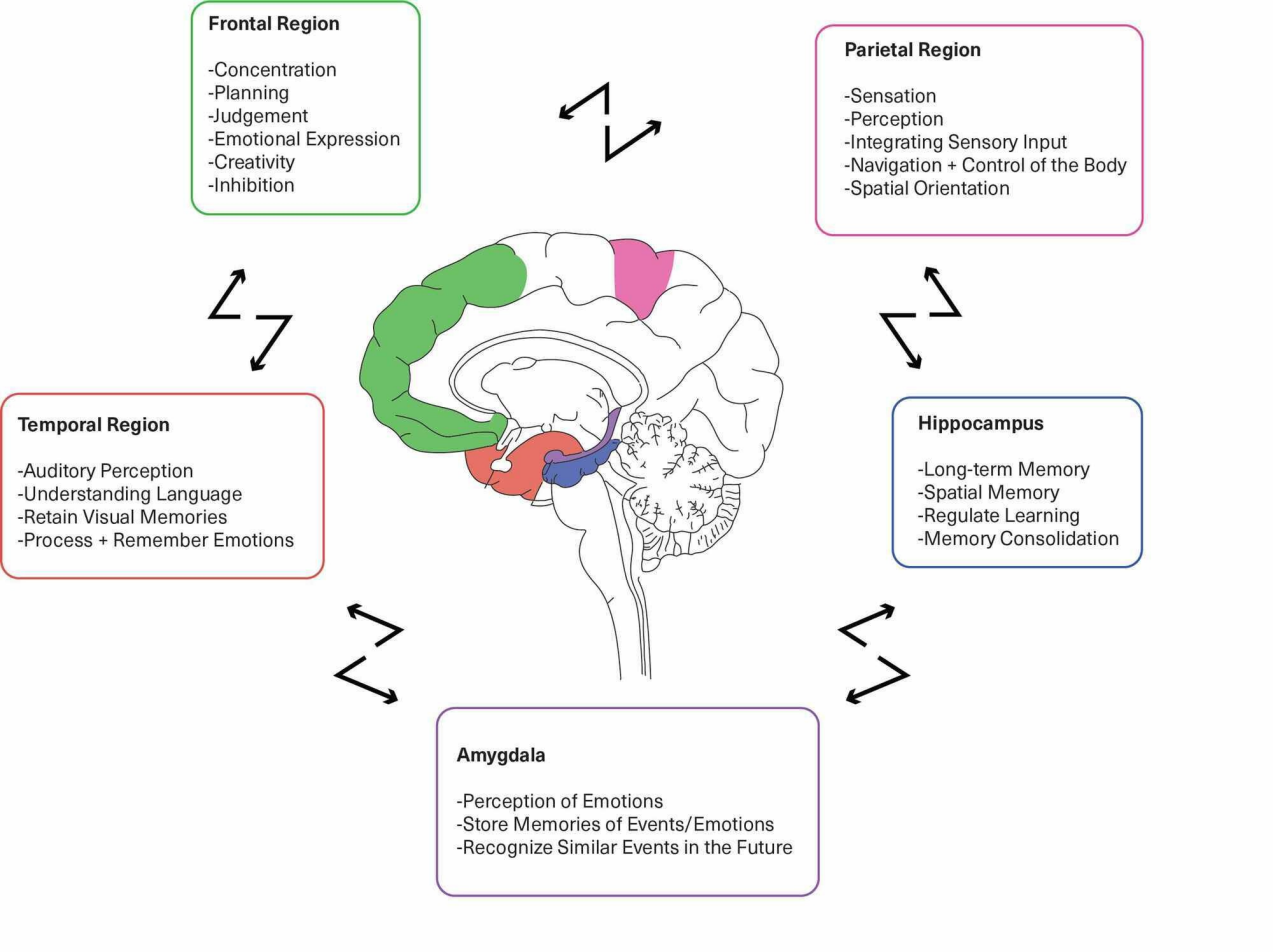
Regions affected by bipolar disorder The figure illustrates the regions affected in BD and their normal functions. An inability of the same areas to connect and collaborate results in the impairment of functions, signified by the broken arrows, mentioned in the model above is expressed in BD.

Neuroplasticity, the production of new brain cells, or restructuring the existing neural circuits induced by various factors have shown to strengthen connectivity in a BD brain. Lithium induces neurotrophic and neuroprotective effects; mindfulness-based cognitive therapy (MBCT) improves mindfulness and emotion regulation, while magnetic seizure therapy decreases cortical inhibition as physical activity enhances happiness, calmness, and higher cognitive functioning. Substantiating neuroplasticity shows excellent promise in redesigning brains toward a learned resilience. Study quality was assessed using the Newcastle Ottawa Scale assessment tool and the Cochrane risk-of-bias tool. After careful screening, 48 of the total 65 studies were included in this review. Out of the selected 48 studies, 22 were observational reviews, 19 clinical trials, five comparative studies, and two meta-analyses. Figure 1 exemplifies the various regions of the brain affected in BD. 

Discussion

Neuroplasticity and Physical Activity in Bipolar Disorder

Bipolar disorder is recognized to contain neurocognitive deficits, more so when associated with psychosis compared to BD without psychosis [17,18]. Studies suggest that differential activation and connectivity in the inferior parietal lobe, inferior frontal lobe, and the precuneus in response to emotional stimuli may be construed as risk markers for BD. The correlation between response inhibition and parietal grey matter volume indicates compensation in parietal cortices [19,20]. Simultaneously, over-activation of frontotemporal regions can be correlated with hyper-perfusion of the respective structures during emotion modulation in patients with BD [14]. Loss of resilience in mood disorders precipitated by stressful experiences is a sign to introduce behavioral and pharmacological intervention [21]. Structural and functional plasticity in the prefrontal cortex (PFC) has shown remarkable ability of the neural circuitry to change based on behavioral experiences, particularly in early childhood and adolescence. For that reason, as illustrated in Figure 1, targeted therapy to the PFC can help strengthen working memory along with self-regulatory and goal-directed behaviors, known to be diluted in BD [21]. Studies show specific neurons contain the extensive capacity for synaptic plasticity due to the continually shifting and holding pertinent details in the working memory until a particular response is warranted [21].

Neurogenesis can enhance the severely lacking executive function in BD, thereby first learning then implementing behavior rules which later lead to success then subsequently modifying them. When repeated stress, 21 days of chronic restraint stress (CRS) was enforced, dendritic shortening in the medial prefrontal cortex was noted while the basolateral amygdala and orbitofrontal cortex showed dendritic growth [21]. This finding illustrates the imbalanced neural growth due to stress on the brain, i.e., in bipolar disorder, which consequently translates into marked impairment in fundamental action prediction, reaction, planning, execution, and verbal memory [12,22,23]. Human Cathepsin B (CTSB) gene expression in the hippocampus has been shown to enhance spatial memory retention and adult neurogenesis. Running induces hypoxia, which elevates hippocampal CTSB expression, in turn promoting clearance of neural debris and adult neurogenesis, thereby potentiating memory and executive function. Doublecortin (DCX) and Brain-derived neurotrophic factor (BDNF) levels are also increased along with the expression of the brain's CTSB gene. BDNF regulates synaptic plasticity, cell survival, and differentiation, as DCX is responsible for neuronal migration [24]. The above mechanisms support the argument of physical activity enhancing the structural and functional stability of a healthy brain and a brain implicated with bipolar disorder. New neurons add a dynamic plasticity level as they undergo increased synaptic plasticity and an enhanced long-term potentiation (LTP) quantified in the dentate gyrus. The new neurons have been shown to avoid interference of new memories with older, similar memories and, as a consequence, promote lasting changes in the network [3]. As physical activity enhances neurogenesis, it has also shown to strengthen immune function, stress regulation, antioxidant defense, circadian rhythms, epigenetic modifications, neurotrophic factors, and telomere maintenance length [1]. This cumulative effect would therefore increase the connectivity within the executive control network, balance the dendritic growth in the prefrontal cortex and amygdala, and even replenish the neurocognitive deficits caused by BD's effect on the parietal lobe, exhibited by reductions in PL white matter volume [11]. Analysis of these investigations reveals that physical activity can produce antidepressant effects on the brain and improve overall cognitive function and quality of life in patients with BD. Some of the included studies focusing on neuroplasticity and physical activity in BD are summarized in Table 1.

**Table 1:** Studies focusing on neuroplasticity and physical activity in bipolar disorder IPL, inferior parietal lobe; IFG, inferior frontal gyrus; ECN, executive connectivity network; PA, physical activity; MCCB, MATRICS Consensus Cognitive Battery; MDD, major depressive disorder; BD, bipolar disorder; PL, parietal lobe; CTSB, Human Cathepsin B; WM, white matter; PFC, prefrontal cortex; PC, parietal cortex; DSM IV, diagnostic and statistical manual of mental disorders fourth edition

Author	Year of publication	Purpose of the study	Intervention studied	Result/conclusion
Nimarko et al. [19]	2019	Emotion processing in mood disorders	Emotion processing	Connectivity between IPL, IFG, and precuneus in association with emotional stimulants may represent resilience and can be used as a risk marker for mood disorders.
Ellard et al. [12]	2018	Connectivity between anterior insula and functional networks in bipolar depression vs. unipolar depression	Functional connectivity	Impaired functional connectivity in ECN in bipolar depression.
Phillips [1]	2017	Physical activity and neuroplasticity in major depressive and BD	Physical activity	Moderate PA may improve the neurobiological and behavioral impairments associated with MDD and BD.
Ferro et al. [11]	2017	Parietal lobe anatomy in BD	White matter reductions	PL WM reductions seem to predict impairment in general functioning in BD.
Moon et al. [24]	2016	Running-induced cathepsin B secretion's association with memory	Running increasing CTSB gene levels	In humans, CTSB gene level changes showed an association between fitness and hippocampus memory function.
Sperry et al. [17]	2015	Cognition in BD with psychosis	Measuring cognition	MCCB can appropriately measure neurocognition but not social cognitive deficits in BD with psychosis.
McEwen et al. [21]	2013	Vulnerability and plasticity of the PFC	Targeted intervention	Studies outline a probable association between the change in brain architecture and improvement in cognitive function and self-regulation.
Teixeira et al. [22]	2014	Integrative PC processes: neurological and psychiatric aspects	PC processes	PC is affiliated with different motor functions, neurological and psychiatric disorders.
Kempermann et al. [3]	2010	Physical activity promotes brain plasticity	Physical activity	Physical activity enhances the assemblage of cells after cognitive stimulation.
Simonsen et al. [18]	2009	Neurocognitive dysfunction in bipolar and schizophrenia	Neurocognitive dysfunction	Neurocognitive dysfunction in bipolar and schizophrenia is decided by the history of psychosis more than the DSM IV diagnostic criteria.
Agarwal et al. [14]	2008	Frontotemporal perfusion in BD	Cerebral blood volume	Frontotemporal hyperperfusion, in bipolar disorder, causes overactivation of these structures during emotional situations.
Haldane et al. [20]	2008	Structural brain response to inhibition in BD type I	Response inhibition	Study suggestive of PFC dysfunction and compensatory involvement of the parietal cortices in BD type I.
Robinson et al. [23]	2006	Cognitive deficits in euthymic BD patients	Cognitive deficits	Euthymic bipolar patients exhibit impaired executive function and verbal memory.

Neuroplasticity and Cognitive Therapy in Bipolar Disorder

**Figure 2:**
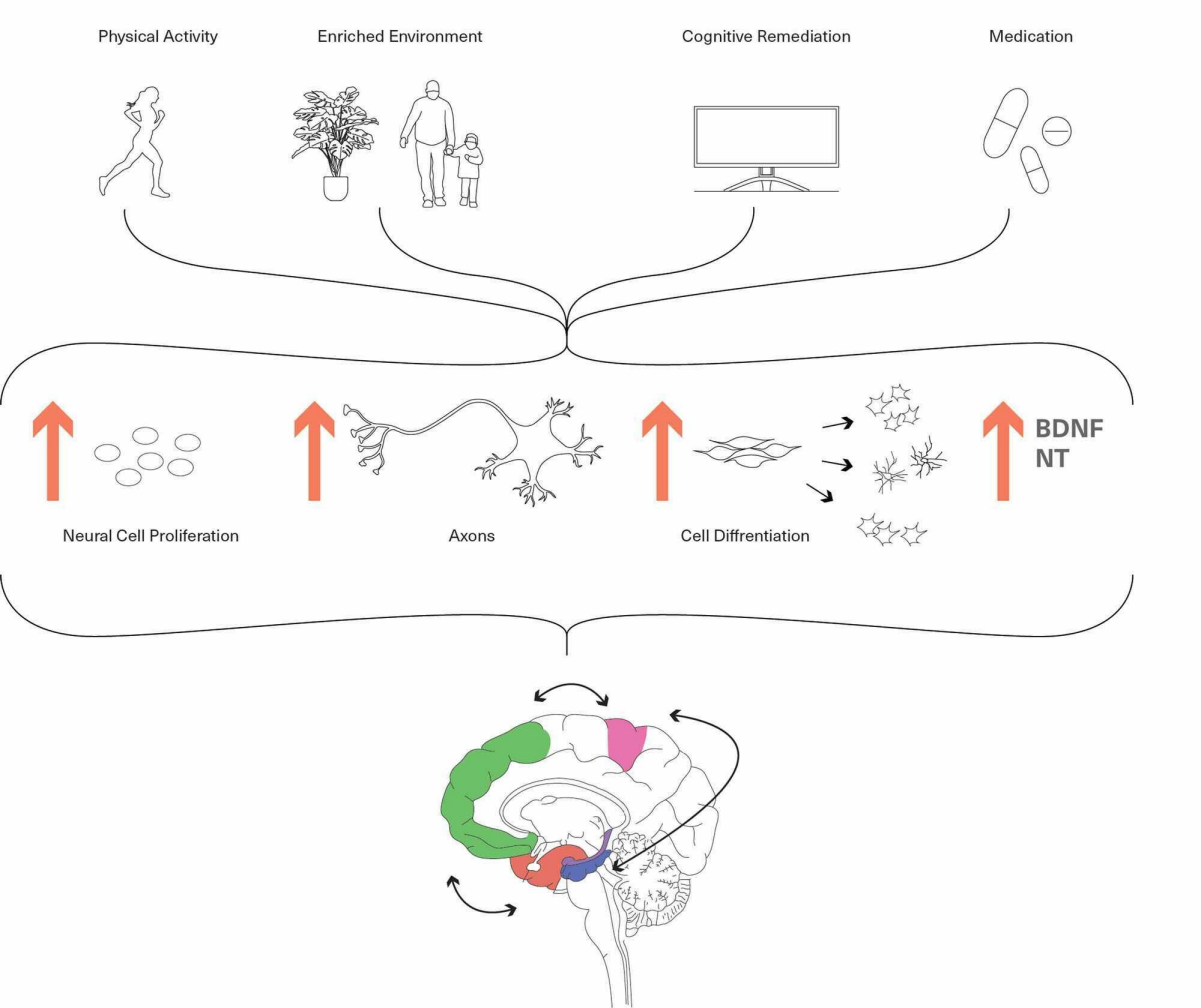
Effects of neuroplasticity in bipolar disorder The figure demonstrates the various mechanisms through which neuroplasticity can be induced, which can later enhance the function and connectivity of the regions affected in a BD brain. BDNF, brain-derived neurotrophic factor; NT, neurotransmitter

Donald Hebb studied rats in different environments to explore the impact of experience and discovered positive long-term effects on learning and memory with enriched environments [3]. Every talent is a learned skill. Every success story involves overcoming adversity, which means the brain developed new neurons and synapses and strengthened the existing to solve the previously non-existing challenge. Uncertainty provides novelty; in other words, a cognitive stimulus. Veyrac, along with his colleagues, demonstrated the existence of olfactory neurogenesis in mice after exposure to novelty odors, as well as enhanced responsiveness in human brains, measured via event-related potential in the electroencephalograph (EEG) showed enhanced capabilities on neuropsychological tests, mainly related to attention and executive functioning [3]. This study is a strong indication that the impaired integration of emotion and memory in bipolar disorder can be strengthened, if not corrected entirely, with consistent exposure to an enriched environment [25]. Lambert et al. and Rosenzweig et al. made a theoretical association between experience and brain structure alterations by illustrating the brain's anatomic and physiologic influences from an enriched environment [26,27]. In BD, the parietal lobe is cardinal to determine spatial sense, information navigation, and integration [22]. The environmental enrichment allows the expression of a significantly more comprehensive range of spatial and social behaviors, significantly impacting long-term memory and learning new skills. In 1997, a link between neurogenesis and hippocampal function was established when new hippocampal granule cells, which then travel to other brain structures, were found in mice living in an enriched environment [3]. Diet, stress, and exercise directly impact neural stem cells, but Valero et al. modulate its indirect neurogenesis effect on microglia [28]. Microglia, the brain's guardian, plays a critical role in maintaining cognitive reserve in the brain while preventing anxiogenic responses that may surface during disease or injury through adulthood and aging [5,28]. Increased neurogenesis on the protective microglia may attenuate the functional connectivity of the executive control network seen in BD (Figure 2). Many got enough time for a daily walk from their busy schedule during the unprecedented time of COVID-19, as workplaces were closed, and most of the population was predominantly indoors. The daily walk in this particular example represents repetitive and consistent behavior, was shown to increase serotonin production in the brain. The joyous anticipation for that daily walk, then increased dopamine production. When including friends in the activity, oxytocin was released, in addition to decreased stress hormones. This entire process encourages neurogenesis as it potentiates the ECN while strengthening emotional resilience and global cognition, known to be impaired in BD [29]. Consequently, the increased potentials on EEG as a result of physical activity combined with the connectivity-enhancing impact of environmental enrichment may reveal a more significant outcome while attempting to strengthen a BD brain. 

In addition to preventing recurrence in bipolar disorder types I and II, group psychoeducation adjunct to pharmacologic treatment are shown to have more significant benefits than either therapy alone [30,31]. Among others, BD is characterized by an imbalance between cognitive control and emotional processing systems, which seem to persist even in euthymic periods. A 2013 study modulated increased activity of the inferior frontal gyri and decreased activity of the right hippocampus and parahippocampal gyrus in response to psychoeducation in euthymic bipolar patients [32], which improves the imbalance by potentiating cognitive control and modulating emotional fluctuations [33]. Increased activations in the medial prefrontal cortex, a principal region of pathophysiology in BD, associated with cognitive flexibility, showed improvement in mindfulness and emotion regulation in response to mindfulness-based cognitive therapy as it potentiates experience-induced plasticity [34,35]. The effort-based reward group showed greater appetitive problem-solving skills in comparison to the non-contingent rats [36,37]. Simultaneously, computer-assisted cognitive remediation, in human adolescents with psychosis or adolescents at high risk, illustrated more effectively trained cognitive ability than computer games [38]. These studies signify that skill acquisition strengthens associations between effort and rewards by promoting neurogenesis in the fronto-parieto-temporal complex leading to increased persistence in an unsolvable task, attentional processing, visuospatial abilities as well as psychosocial functioning, which are impaired in BD [38,13]. Schizophrenia and BD both have shown overlapping impairments in global cognition, attentional processing, social functioning, and cognitive flexibility [10]. Substantial improvements in the above categories have been revealed in schizophrenic subjects receiving active training and enhanced self-esteem with cognitive remediation [39,40]. It would, therefore, be a fair assumption to expect similar neuroplastic effects in patients with BD (Figure 2). BD is construed as chronic stress by the brain. In response to sudden changes, i.e., stress, neurons apply homeostatic mechanisms to maintain proper function and initiate experience-dependent plasticity. Certain axonal arbors, boutons, and dendritic spines appear and disappear, leading to synapse formation and elimination [41,42]. This process enhances emotional and neurobiological resilience; the very abilities weakened in bipolar disorder.

Furthermore, Lambert et al. revealed not only effort-based reward training, but the group of rats with flexible coping mechanisms showed enhanced neuroplasticity more consistently than the active and passive copers [9]. Thus, cognitive remediation via various mechanisms 'made' new cells can potentially alter the approach to bipolar disorder therapy. Table 2 shows some of the included studies.

**Table 2:** Studies on cognitive remediation BD, bipolar disorder; EBR, effort-based reward; CACR, computer-assisted cognitive remediation; MBCT, mindfulness-based cognitive therapy; PFC, prefrontal cortex; EBP, euthymic bipolar patients; PC, parietal cortex

Author	Year of publication	Purpose of the study	Intervention studied	Result/conclusion
Jamann et al. [42]	2018	Activity-dependent axonal plasticity	Somatodendritic domain and axonal domain	Presynaptic changes induce homeostatic regulation of excitability during development in an experience-dependent manner in adults.
Favre et al. [33]	2016	White matter plasticity and psychoeducation in BD	Psychoeducation	Psychoeducation may be connected to improved emotional regulation in BD.
Valero et al. [28]	2016	Adult neurogenesis and lifestyle correlation	Lifestyle and environment	Adult hippocampal and microglial neurogenesis is modulated by the demands of the environment and lifestyle factors.
Lambert et al. [5]	2016	Neurobiological resilience by the environment	Natural-enriched environments	Natural-enriched rats exhibited less anxiety-typical behavior.
Shaffer [29]	2016	Neuroplasticity and clinical practice	Positive behavioral techniques	Positive behavioral techniques could improve the global statistics on cognition.
Zhang et al. [25]	2015	Brain activation during reflection in BD	Reflection	This study shows impaired connectivity between emotion and memory in patients with BD.
Lambert et al. [26]	2015	Neurobiological effects of urbanization	Urban vs. rural habitat	Urban habitats compared to rural may lead to increased vulnerabilities for maladaptive neurobiological functions and the consequent emergence of psychiatric illness.
Lambert et al. [9]	2014	Emotional resilience based on circumstance	EBR training	EBR training showed neurobiological resilience with adaptive responses to prediction errors.
Laurent [38]	2014	CACR in psychosis	CACR	CACR group showed a more significant improvement in visuospatial abilities than in cognitive functions.
Fuchs et al. [13]	2014	Adult neuroplasticity	Adult neuroplasticity	Regulated adult neurogenesis can be used for future therapeutic interventions.
Bardi et al. [37]	2013	EBR training in a problem-solving task	EBR training	EBR training potentiates cognitive function and emotional modulation in exacting situations.
Ives-Deliperi et al. [34]	2013	MBCT in BD	MBCT	MBCT in BD improves mindfulness, emotion regulation, reduces anxiety, and enhances activation in the PFC, an area responsible for cognitive flexibility.
Favre et al. [32]	2013	Psychoeducation in EBP	Psychoeducation	Psychoeducation enhances cognitive control and modulates emotional fluctuations in EBP.
Teixeira et al. [22]	2013	Integrative parietal cortex processes: neurological and psychiatric aspects	Parietal cortex processes	PC is affiliated with different motor functions, neurological and psychiatric disorders.
Bardi et al. [36]	2012	If adaptive behavioral training builds resilience against stress-induced pathology	Effort-based reward training	EBR rats persisted longer in appetitive problem-solving tasks.
Dayan and Cohen [35]	2011	Neuroplasticity subserving motor skill learning	Motor skill learning	Learning-induced structural changes in gray and white matter.
Kempermann et al. [3]	2010	Physical activity promotes brain plasticity	Physical activity	Physical activity increases cognitive stimulation.
Holtmaat and Svoboda [41]	2009	Experience-dependent structural synaptic plasticity	Sensory experience and learning	Synapse formation and elimination promotes experience-dependent rewiring of neural connections.
Fisher et al. [39]	2009	Neuroscience of learning-induced neuroplasticity	Neuroplasticity-based auditory training	Active training resulted in significant gains in global cognition, verbal working and learning memory.
Colom et al. [30]	2008	Psychoeducation for BD type II	Psychoeducation	Psychoeducation plus medication can benefit BD type II subjects.
O'Donnell et al. [10]	2004	Auditory abnormalities in BD and schizophrenia	Auditory processing	This study showed impaired attentional processing in schizophrenia and symptomatic BD.
Colom et al. [31]	2003	Role of psychoeducation in patients with BD	Group psychoeducation	Group psychoeducation can be used to prevent recurrence in BD types I and II.
Wykes et al. [40]	1999	Neurocognitive remediation on executive processing in schizophrenia	Neurocognitive remediation	Cognitive remediation can reduce cognitive deficits.
Rosenzweig and Bennett [27]	1996	Effects of training and experience	Training or differential experience	Enriched early experience improved performance on several learning tests.

Neuroplasticity and Medical Therapy in Bipolar Disorder

Lithium is the only consistently used mood stabilizer for acute mood episodes, prophylactic therapy, and suicide prevention in BD. It is the neurotrophic effects that help cell proliferation and regeneration, while the neuroprotective effects limit neuronal atrophy and cell death. These effects are greatly pronounced in the presence of pathology, following the onset of BD [43,44]. Lithium is also known to modulate apoptosis and influence the ameliorating effects of excitotoxicity [4,45]. BDNF is known to enhance neuronal maturation, differentiation, and survival, synaptic plasticity, long-term memory consolidation, and is distinctly expressed in the cerebral cortex and hippocampus. Studies have reported decreased BDNF levels in BD, and BDNF is also known to play a role in BD progression and treatment response. Chronic lithium treatment has been shown to upregulate BDNF levels [4,2]. An increase in BDNF levels has revealed decreased depressive and manic episodes, suggesting the use of serum BDNF as a potential biomarker in BD [46]. The increase in neuroplasticity by lithium attenuates the decrease in both grey and white matter in patients with BD [4], even possibly emend the BD-induced visuospatial asymmetry [47]. While anodal transcranial direct current stimulation is associated with neuroplastic effects in stroke patients, BD's effect is yet to be explored [48]. While, in theory, repetitive transcranial magnetic stimulation in humans is a promising non-invasive method of stimulating neurogenesis, its effect on synaptic plasticity is reported to be weak and rarely lasting longer than 30 minutes [6]. However, not part of the routine protocol, magnetic seizure therapy is another method explored to decrease suicidal ideation. MST has shown to enhance neuroplasticity in the frontal cortex, showing a concomitant decrease in cortical inhibition by stimulating LTP-like plasticity [7]. Even though the discovery of astrocyte plasticity is profound, its contribution to neuronal plasticity remains unclear and requires further research [8]. Table 3 shows some of the studies discussing neuroplasticity and medical therapy.

**Table 3:** Studies on neuroplasticity and medical therapy MST, magnetic seizure therapy; tDCS, transcranial direct current stimulation; BD, bipolar disorder; BDNF, brain-derived neurotrophic factor

Author	Year of publication	Publication of study	Intervention studied	Result/conclusion
Sun et al. [7]	2018	MST's effect on suicidal ideation and neuroplasticity	Magnetic seizure therapy	MST produced improvement in suicidal ideation and neuroplasticity in the frontal cortex.
Hordacre et al. [48]	2018	Neuroplasticity following a stroke	tDCS	Sensorimotor and motor‐premotor network is a biomarker of neuroplastic induction following anodal tDCS in chronic stroke survivors
Machado-Vieira [44]	2017	Lithium, stress, and resilience in BD	Lithium	Lithium induces neurotrophic and neuroprotective effects in BD.
Won and Kim [4]	2017	Lithium in the treatment of BD	Lithium	Although lithium is an undeniable treatment and prophylaxis option in BD, not all patients benefit from it.
Sigitova et al. [2]	2016	Biomarkers of BD	Antidepressants	BD may be a result of neural damage, and antidepressants may increase neural and cortical plasticity.
Sims et al. [8]	2015	Correlation of astrocyte and neuronal plasticity with the somatosensory system	Astrocyte plasticity	Although astrocyte's roles in the barrel cortex are established, the possible interactions of astrocyte plasticity are unknown.
Najt et al. [47]	2013	Spatial attention in BD	Right frontoparietal dysfunction	Impaired functional cerebral symmetry is noted in euthymic BD patients in regards to visuospatial attention.
Soeiro-de-Souza et al. [43]	2012	Translating neurotrophic and cellular plasticity for BD	Neurotrophic factors	Treatment with mood stabilizers, specifically lithium, re-establishes pathophysiological changes in BD.
Machado-Vieira et al. [45]	2009	Lithium in the treatment of the BD	Lithium	Lithium corresponds to significant neurotrophic property changes not only in BD but also in other brain and neurological disorders.
Kapczinski et al. [46]	2008	The BDNF factor and neuroplasticity in BD	BDNF	BDNF plays a pivotal role in the manifestation of psychosocial stress and recurrent manic episodes in BD.
Huang et al. [6]	2005	Stimulation of the human overstimulation	Theta burst stimulation	The method may prove useful in the motor cortex and other regions of the brain for normal human physiology and the therapeutic manipulation of brain plasticity.

Limitations

Due to the lack of comprehensive research specific to the correlation between neuroplasticity and bipolar disorder, its impact on the current BD treatment protocol is limited to mood stabilizers like lithium. Therefore, it is encouraged to study further the impact of various forms of neuroplasticity in BD in a clinical environment. 

## Conclusions

Compromised integrity of the frontosubcortical and prefrontolimbic brain regions contribute to the depressed mood and impaired cognitive coping in BD. Excessive activation in the brain has been associated with emotional regulation and contributes to BD's affective symptoms, with a predominant effect on the hippocampal circuitry. The purpose of neurogenesis in BD is to increase the number of precursor cells, enhancing cell proliferation and increasing axonal connectivity. Physical activity induces hippocampal neurogenesis and may contribute to the reversal of BD associated emotional and cognitive deficits, especially when paired with various forms of cognitive therapy. Cognitive therapy, along with physical activity, can strengthen the ECN while significantly changing the progression of BD. Alongside medical therapy in BD, if a predetermined program is created, implementing regular physical activity and cognitive therapy, possibly earlier on in the course of this disorder, an exponential decrease in BD progression with significantly improved quality of life can be predicted. While lithium is already proven for its neurotrophic and neuroprotective effects, the role of physical activity and cognitive therapy in the treatment of BD requires further research. 
